# Positive relationships among aboveground biomass, tree species diversity, and urban greening management in tropical coastal city of Haikou

**DOI:** 10.1002/ece3.7985

**Published:** 2021-08-05

**Authors:** Mir Muhammad Nizamani, AJ Harris, Xia‐Lan Cheng, Zhi‐Xin Zhu, Chi Yung Jim, Hua‐Feng Wang

**Affiliations:** ^1^ Key Laboratory of Tropical Biological Resources of Ministry of Education College of Tropical Crops Hainan University Haikou China; ^2^ Key Laboratory of Plant Resources Conservation and Sustainable Utilization South China Botanical Garden Chinese Academy of Science Guangzhou China; ^3^ Department of Social Sciences Education University of Hong Kong Hong Kong China

**Keywords:** ecosystem services, functional traits, multiple regression, Simpson diversity index (*D*)

## Abstract

Within urban green spaces, tree species diversity is believed to correlate with aboveground biomass, though there is some disagreement within the literature on the strength and directionality of the relationship. Therefore, we assessed the relationship between the biodiversity of woody species and the aboveground biomass of woody plant species in the tropical, coastal city of Haikou in southern China. To accomplish this, we obtained comprehensive tree and site data through field sampling of 190 urban functional units (UFUs, or work units) corresponding to six types of land uses governmental‐institutional, industrial‐commercial, park‐recreational, residential, transport infrastructure, and undeveloped area. Based on our field data, we investigated the relationship between tree species diversity and aboveground biomass using multiple regression, which revealed significant relationships across all five types of land uses. Aboveground biomass in green spaces was also correlated with anthropogenic factors, especially time since urban development, or site age, annual maintenance frequency by human caretakers, and human population density. Among these factors, maintenance is the strongest predictor of aboveground biomass in urban green space. Therefore, this study highlights the critical role of maintenance of urban green space in promoting both aboveground biomass and woody biodiversity in urban ecosystems and, consequently, on urban ecosystem services. Our findings contribute to a deeper understanding of the ecosystem services provided by communities of woody plant species in urban areas.

## Graphical abstract

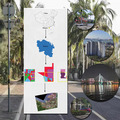

## 1 INTRODUCTION

Ecosystems provide many services that are beneficial to humans, and ecosystem services are typically more wide‐ranging when there is greater biodiversity (Dobbs et al., 2011). Ecosystem services provided by urban green space may include decreasing local temperatures and improving air quality (European Commission, 2015) as well as limiting some or many of the environmental impacts of landscape clearing that occurs during the development of city infrastructure (Carrus et al., 2015). In ecosystems dominated by woody plant species, services include soil formation and air purification, both of which are also positively correlated with the aboveground productivity of a plant community as a whole (Baró et al., 2014; Grace et al., 2016; Pesola et al., 2017). Aboveground productivity refers to the accumulation of aboveground biomass (AGB), especially of stems and leaves, during annual growing seasons. AGB is correlated with many ecosystem services, and thus, it has a well‐established relationship with biodiversity in natural systems (Zhang et al., 2016). However, the relationship of AGB to tree biodiversity in urban areas remains poorly known (Wilkes et al., 2018).

**FIGURE 1:**
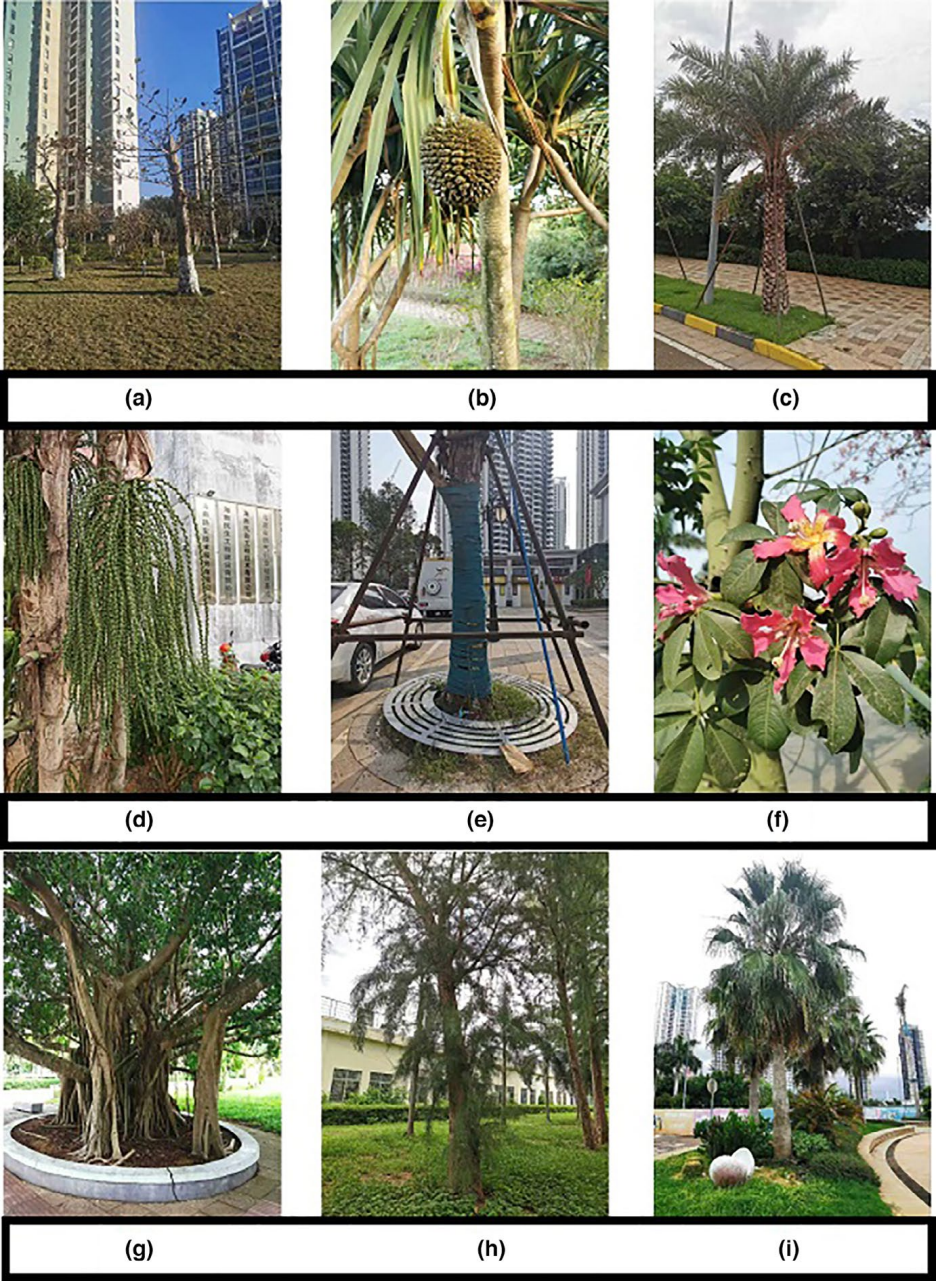
Field sampling plot showing the urban forest of Haikou. (a) *Terminalia catappa* (Gaertn.) Eichler in the residential area of Haikou, the top of their trunk was lost in the typhoon. (b*) Pandanus utilis* Bory. (c) *Phoenix dactylifera* L. (d) *Roystonea regia* L.H. Bailey. (e) *Caryota mitis* Lour. (f) *Ceiba speciosa* (A. St.‐Hil.) Ravenna. (g) *Ficus benghalensis* L. (h) *Casuarina cunninghamiana* Miq. (i) *Washingtonia robusta* H. Wendl

Within cities or high‐density urban areas, ecosystem services benefit from the species richness harbored in urban green spaces (da Silva et al., 2021; Kowarik et al., 2019). Urban green spaces consist of native and non‐native species and species that are spontaneous or cultivated within a city and its surrounding areas (Chang et al., 2021; Palliwoda et al., 2017), but woody species, in particular, mitigate climate change, carbon saturation and improve air quality (Khedive et al., 2017; Sahle et al., 2018). The woody component of urban green space (Figure 1; hereafter, “urban green space”, but referring specifically to the woody component) can be defined as perennials, especially trees of all types, such as dicots, monocots, lianas, gymnosperms, pteridophytes, and others that may be aggregated within forests or orchards or represent a few or isolated individuals, such as street trees (Salbitano et al., 2016). In general, urban green spaces are managed ecosystems and rely on regular human input. Within urban green spaces, the biomass of species is controlled via silvicultural practices including thinning, logging, selective cutting, and enrichment planting (Verschuyl et al., 2011).

Cities are often regarded as centers of high plant diversity with an especially large number of non‐native species (Ignatieva, 2010; Müller & Kelcey, 2011; Wang & López‐Pujol, 2015). To explain the high levels of biodiversity in cities, several nonmutually exclusive hypotheses have been developed. One hypothesis is the "luxury effect" (Hope et al., 2003), which suggests that biodiversity is dependent on the affluence of an urban area, with greater diversity in more affluent areas (Leong et al., 2018). Another hypothesis concerns the age of urban areas, or site age, with older areas having greater diversity due to the accumulation of species introduced by humans intentionally or accidentally or that establish spontaneously (Luck et al., 2009; Troy et al., 2007; Wang et al., 2020). A third hypothesis concerns the frequency of management of urban green space, with areas undergoing more frequent management by human caretakers having higher diversity due to the influx of resources like water and fertilizer (Battie‐Laclau et al., 2016). However, these potential predictors of tree species diversity within urban green spaces remain incompletely understood, and, in particular, their relationship to the AGB of woody species is underexplored. Moreover, urbanization in coastal cities is frequently linked to rapid changes in how land is used, and changes in land use can also potentially transform green space (Mansour et al., 2020; Zhang et al., 2020; Zhu, Pei et al., 2019). Thus, understanding how land use drives the AGB of woody species is potentially valuable for planning and maintaining urban green space.

Among cities, coastal cities are often older than interior ones as modern humans have largely preferred (or been strategically more likely) to settle along coasts (Gillis & Barringer, 2012). Moreover, coastal cities are also typically more expensive places to live and attract residents with greater disposal income (Zhang & Chen, 2015). In recent years, the world's coastal cities have experienced substantial, rapid growth in both population and urban development (Neumann et al., 2015) and simultaneously attracted increasingly large numbers of tourists (Liu et al., 2017). Thus, the long history of coastal cities and their tendency to attract both affluent residents and tourists may yield increased diversity of tree species as predicted by both the luxury effect and site age hypotheses.

In this study, we investigated the relationship between tree species diversity and AGB in the tropical, coastal city of Haikou in China. We sought to address the following questions: (1) How does AGB of woody species vary among types of land use within Haikou? and (2) What are the drivers of AGB within the urban types of land use? For drivers of AGB, we examined tree species diversity as well as anthropogenic factors known to be linked to the major hypotheses of distributions of urban green space including maintenance frequency, site age, and population density.

## 2 METHODS

### 2.1 Study area

The extent of our study area comprised the city of Haikou (20° N and 110° E), which is a coastal city and the capital of the southernmost province in China, the island of Hainan. Haikou is located on the northern coast of Hainan and is the political, cultural, and economic center of the province. Haikou emerged as an urban area ca. 2000 years ago during the Han dynasty (200 BC) and was fortified as a military post by the Ming dynasty in the 13th century (Swope, 2014). Prior to urbanization, the region was inhabited by the indigenous Li, who are descendants of the first human groups that reached the island seven to 27 thousand years ago (Peng et al., 2011). Haikou was developed as a coastal port during WWII when the Japanese occupied Hainan Island from 1939 to 1945 (Lary, 2010). Within this study, we defined the boundaries of Haikou using two main sources: Haikou People's Government (2011) and Cox (2015).

At present, Haikou comprises a major coastal port city at the mouth of the Nandujiang River and covers ca. 3,146 km^2^. Within Haikou, there are approximately 284 km^2^ of urban green space and 861.44 km^2^ of nonforested land (Hainan Bureau of Statistics, 2014). For purposes of this study, nonforested land refers to the managed land area within the city that is developed for purposes other than for the production of timber or various forest products or the maintenance of woody vegetation, such as in protected areas (Chazdon et al., 2016). Of the forested land area of Haikou, approximately 95,800 ha, or 42%, comprises tropical economic crops, such as rubber trees, oil palms, and coconuts (Hainan Bureau of Statistics, 2014).

The climate of Haikou is typical of tropical areas in that it is frost‐free. The city experiences fog in the spring, thunderstorms in the summer, typhoons in the autumn, and cool, dry weather in the winter (Haikou Meteorological Bureau, 2019 **)**. The average annual temperature is 23.8°C, and the city receives 1,639 mm of precipitation per year (Haikou Meteorological Bureau, 2019 **)**. The tropical climate of Haikou supports many native and non‐native plant species including 1980 terrestrial plants, of which more than 40 are endemic to Hainan (Zhu, Pei et al., 2019). Several native and cultivated species are nationally protected, such as *Cycas revoluta* Thunberg, *Dalbergia odorifera* T. Chen and Thunb., and *Hopea hainanensis* Merr. et Chun, which are Class I protected species within China, and *Antiaris toxicaria* Lesch, *Aquilaria sinensis* (Lour.) Spreng, *Cephalotaxus sinensis* (Rehd. et Wils.) Li, and *Dalbergia hupeana* Hance, which are Class II protected species (Zhu, Pei et al., 2019).

### 2.2 Sampling design and remote sensing image interpretation

#### 2.2.1 Land use definitions in Haikou

In order to compare AGB and species diversity among types of land use in Haikou, we divided land uses into six standard categories: industrial‐commercial, government‐institutional, residential, park‐recreational, transport infrastructure, and undeveloped land following the American Urban Forestry classification system (Anderson, 1976) (Table 1). Industrial‐commercial areas consist primarily of commercial buildings, such as downtown, central business district, financial district, main street, commercial strips, or shopping centers. Government‐institutional refers to a government‐created and/or operated professional entity, such as hospitals, educational institutions, and government facilities. Residential areas are land where housing predominates, while park‐recreational areas cover public property (e.g., nature parks, public walks, outdoor art installations), as well as private amenities (e.g., hotel properties and golf courses). Transportation infrastructure includes railways and rail stations, roads and terminals, airports, port infrastructure, bus stations, gas stations, and warehouses. Undeveloped land has no infrastructure and generally comprises land devoid of woody species (e.g., beaches). We defined these six types of land use according to the American Urban Forestry classification system (Anderson, 1976) but assessed their suitability for application to a Chinese city, especially based on prior studies (Wang et al., 2015, 2016; Zhu, Pei et al., 2019; Zhu, Roeder et al., 2019).

**TABLE 1:** The six standard categories of urban types of land use are evaluated in this study

Land use	Description	Number of sample plots	Sample plots (%)
Government‐institutional	Hospitals, educational facilities, government services.	59	31.05
Industrial‐commercial	Industrial and commercial sites.	35	18.42
Park‐recreational	Green spaces are typically maintained by government agencies.	12	6.32
Residential	A densely populated urban area with single or multiple residential units.	48	25.26
Transport infrastructure	Transportation and utilities.	33	17.37
Undeveloped land	Vacant land and land under construction.	3	1.58
Total		190	100.00

#### 2.2.2 Sampling within Haikou

**FIGURE 2:**
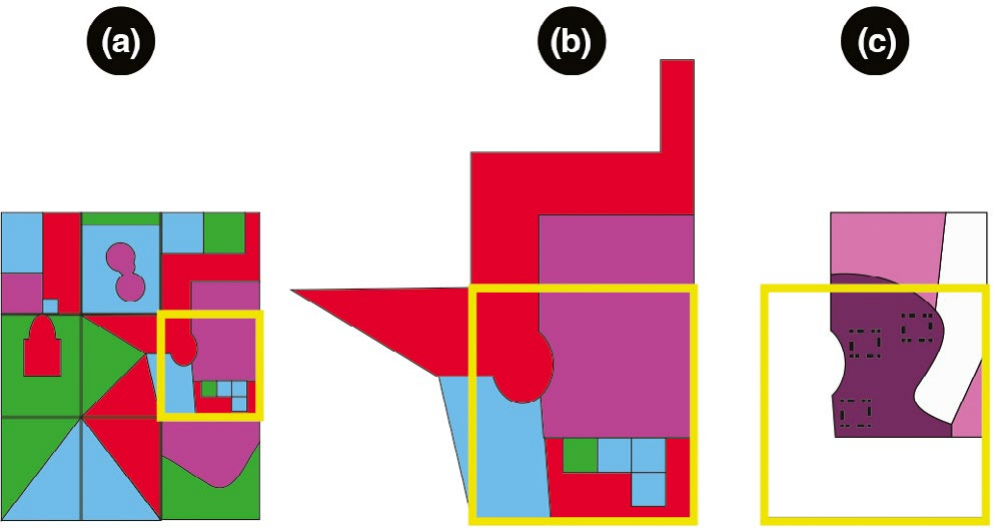
Workflow for establishing urban sampling plots used in this study is shown using a cartoon diagram. (a) We established a grid over the full geographic extent of the study area. Within each grid cell (highlighted in yellow), we identified associated aggregates of infrastructure, or work units, and assigned these to one of four types of land use. In this cartoon, the types of land use comprise Red, Green, Blue, and Purple, and each aggregate (outlined in black) may extend into multiple grid cells. (b) Within each grid cell, such as the one highlighted in yellow, we identified (i) work units occupying the most land area within the cell and (ii) any culturally significant work units, such as parts, historical sites, and so on. (c) Within work units covering the most land area (i.e., Purple) and in culturally significant sites if any (i.e., Blue), we performed fieldwork to visually identify areas suitable for establishing sampling plots (i.e., they contained vegetation, not roads or buildings as indicated by white areas), and we sought to find the most diverse areas for plots (i.e., represented by increasingly dark colors) to best determine the full extent of species diversity within the sampled work unit. Within the sampling plots, we counted all woody species that we observed

**FIGURE 3:**
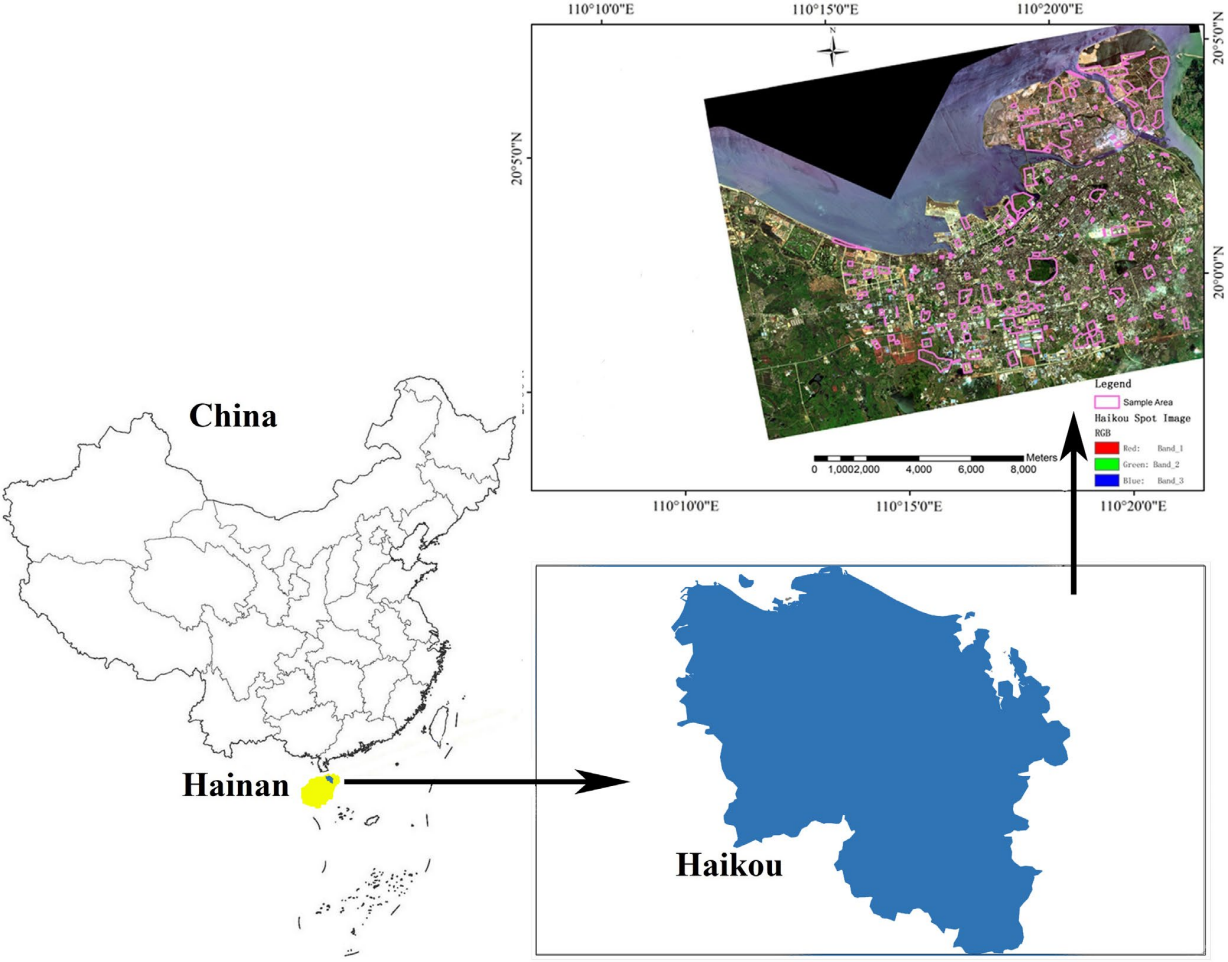
Images showing the locations of the study. (a) Map of China yellow highlighted is Hainan and blue highlighted is Haikou. (b) Map of Haikou. (c) SPOT‐7 satellite image of Haikou city showing the vegetation cover in relation to impermeable area cover and the sample area

We used a three‐step, stratified, representative process (Figure 2) to establish sampling plots within Haikou following prior studies (Wang et al., 2015, 2016, 2020). First, we developed a sampling grid using an image of the city taken in October 2010 via the French satellite, Satellite Pour l'Observation de la Terre (SPOT; https://www.satimagingcorp.com/satellite‐sensors/other‐satellite‐sensors/spot‐7/). We corrected this image orthographically and embedded it using ERDAS IMAGINETM (https://www.hexagongeospatial.com/products/power‐portfolio/erdas‐imagine). We overlaid the image with a grid comprising 190 650m × 650m cells using the OvxitalMap tool (http://www.ovital.com:8080/en/) on the Google Earth satellite view (https://earth.google.com/web) (Figure 3). Thereafter, within each cell, we selected one representative urban functional unit (UFUs, e.g., Hainan University or Haikou Renmin Hospital). We chose the representative UFUs based on the characteristics of each grid. Most often, we determined the most pervasive type of land use within a cell and selected a UFU representing that type of land use. For example, within a cell comprised mostly of industrial‐commercial area, we choose one company as the representative UFUs. However, sometimes work units that were particularly culturally or economically significant were selected, such as Hairui Tomb, even if their type of land use was not the most pervasive within the grid. Within the area of each selected, representative UFU, we established at least three 20 m × 20 m sampling plots for plant diversity and abundance to represent the plant diversity of this UFU. Ideally, we established the plots in areas of the UFUs that were the most diverse, so that we could sample the full range of diversity harbored by the UFU, but establishing plots in areas readily accessible for sampling was also a necessity. This approach to a stratified, representative sampling of urban green spaces was first proposed in Wang et al. (2016) because a randomized sampling design is not suitable for this kind of study, as some randomly selected plots may have been located in inaccessible places or contained no vegetation. All data from the 20 m × 20 m plots within each UFU were merged. In our sampling, we especially sought to ensure that all types of land use in Haikou were sampled roughly proportionally, and we avoided sampling the same plot twice by regarding UFUs are representing only one type of land use each (Wang et al., 2015, 2016). To visualize each cell and UFU, we used ArcGIS10.1 (https://www.esri.com/), and our final selection of UFUs is shown in Figure 3.

### 2.3 Field measurements

**FIGURE 4:**
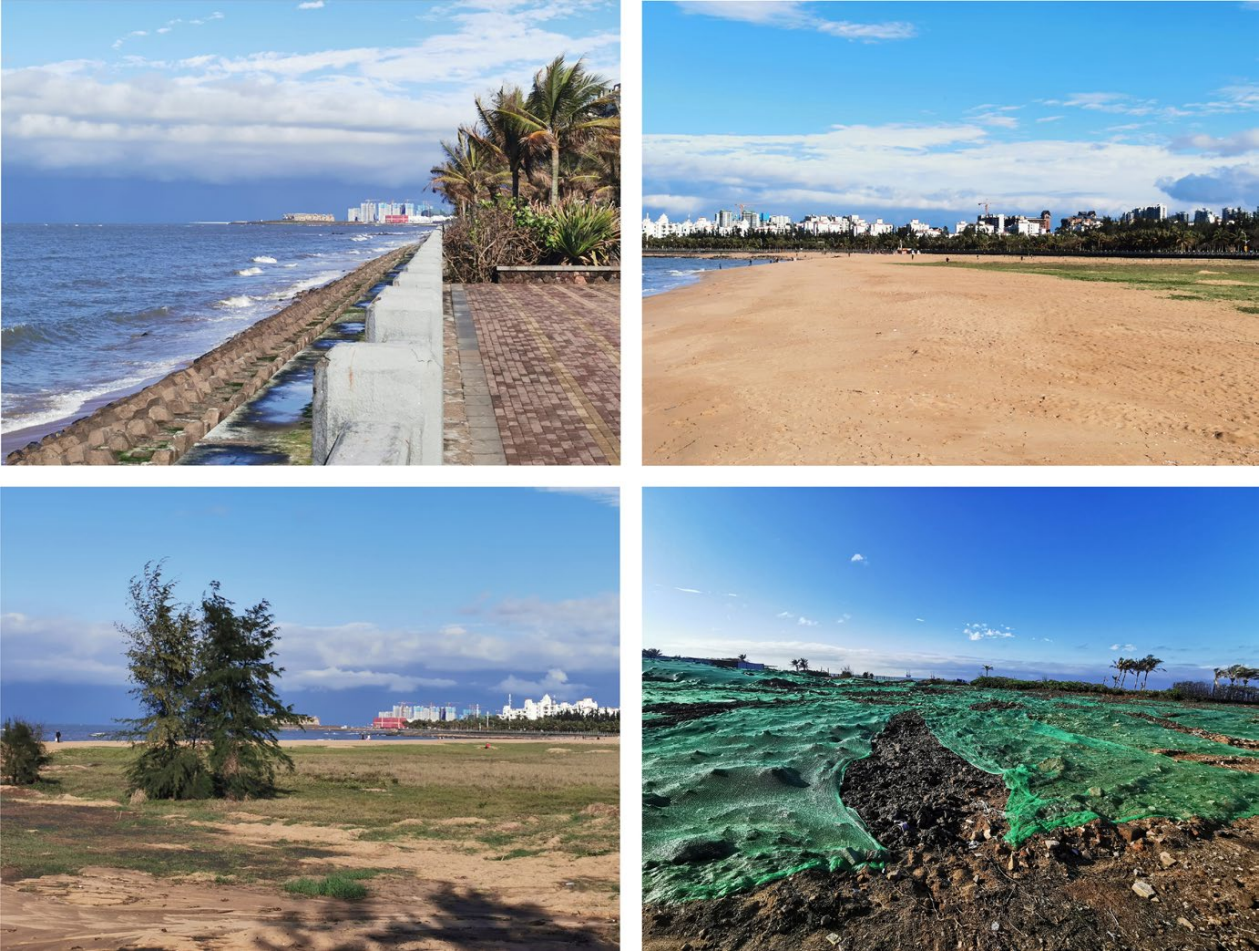
Four pictures of the undeveloped types of land use

In the field plots, we counted the abundance of woody species with a diameter at breast height (DBH; ca. 1.3 m above ground) of ≥2 cm. To identify species, we followed the Flora of China (Flora of China Committee, 2013), and for each woody individual, we recorded the trunk DBH (cm), height (cm), and canopy width. For the UFUs representing the undeveloped type of land use, we found only one, two, or no woody species (e.g., Figure 4), and, therefore, we removed this type of land use from downstream analyses.

### 2.4 Site age determination

In order to measure site age, we used building age, which is commonly regarded as a proxy for site age (Wang et al., 2020). To obtain building age, we first determined the year of a building's construction according to administrative records, when available, or by interviewing at least five maintenance workers (Wang et al., 2015, 2016; Zhang et al., 2016; Zhu, Pei et al., 2019; Zhu, Roeder et al., 2019). We acknowledge that interviews may lead to imprecise estimations of build ages, so we averaged among respondents and observed that there were not orders of magnitude difference among them. For our study, this level of age resolution (i.e., 10s of years old versus 100s versus 1,000s) was suitable. We then subtracted the year of construction from 2019 to obtain the building age. Although the data analyses for this study were performed in 2019, the relative ages among buildings (and, thus, the conclusions of the study) will not change.

### 2.5 Green space management frequency determination

We measured the frequency of management of urban green spaces based on occurrences of fertilization and watering, which we determined by consulting records of the local management departments or, when not feasible, by interviewing at least five management workers per plot. As with interviews regarding building age, we averaged management workers' responses to try and mitigate any misrepresentations of memory.

### 2.6 Population density determination

By visiting each grid cell, we determined the population based on the number of residential buildings (*B*) within it, the number of stories for each building (*S*), and the number of dwelling units on each story (*A*). Additionally, we used the Haikou Statistical Yearbook (Hainan Bureau of Statistics, 2014) to determine the average family size (*M*). Using these parameters, we applied the following formula to determine the population (*P*) for each grid cell. *P* = *B* × *S* × *A* × *M*. Based on this, we inferred population density (people / km^2^) as *P*/Ar, where Ar is the area in km covered by each grid cell. In our case, Ar is typically value km^2^ except on the periphery of the study area, where it was sometimes smaller because the grid cells were truncated.

### 2.7 Indices of the diversity of tree species

The diversity of species in a plant community can be denoted by several key indices (Cai et al., 2013). In this study, we calculated the Simpson diversity index (*D*) (Simpson, 1949), the Shannon diversity index ($H_{e}^{\prime }$) (Shannon, 1948), and Pielou evenness index (Je) (Pielou, 1966). The Simpson diversity index (*D*) refers to the probability that two individuals randomly selected from a plot will belong to different species (Simpson, 1949) and is calculated as follows:

(1) $$D=1‐\sum_{i=1}^{n}Pi^{2}P_{i}^{2}=\frac{n_{i}\left(n_{i}‐1\right)}{N\left(N‐1\right)}$$

The Shannon diversity index ($H_{e}^{\prime }$) is a measure of the local diversity of a plant community (Shannon, 1948) and is determined using the formula:

(2) $$H_{e}^{\prime }=‐\sum_{i=1}^{S}P_{i}\ln P_{i}$$

Pielou's evenness index (Je) refers to the distribution of the total number of individuals in a community or environment (Pielou, 1966) and results from the following calculation:

(3) $$\text{Je}=\frac{H_{e}^{\prime }}{H_{e_{\max}}^{\prime }}$$

In the above equations: *Pi* is equal to *ni*/*N*, where *ni* is the number of individuals of a single tree species (*i*) and *N* is the number of individuals of all tree species. In Pielou evenness index (Je), $H_{e}^{\prime }$ refers to the result from a calculation of the Shannon diversity index ($H_{e}^{\prime }$), while $H_{e_{\max}}^{\prime }$ is equivalent to ln(*S*), representing a calculation of Shannon's diversity index ($H_{e}^{\prime }$) in which every species in the dataset has an equal frequency. We also measured species richness (*S*) for each sampled plot. We calculated these diversity measures for each plot within a UFU and then averaged the results. We also measured species richness (*S*) for each plot and, similarly, combined the results for each UFU.

### 2.8 Calculation of aboveground biomass

We calculated AGB for each woody species with a DBH ≥2 cm in each sampled plot using a formula proposed by Wang and Xu (2017):

(4) $$\text{AGB}=0.4\pi {\left(\frac{\mathrm{dbh}}{2}\right)}^{2}\times \left(\text{height}+300\right)\times \left(\text{wood density}\right)$$

We obtained the wood density parameter from the TRY database (https://www.try‐db.org/TryWeb/Home.php), which is a global database maintained by a network of vegetation scientists and led by Future Earth and the Max Planck Institute for Biogeochemistry (Kattge et al., 2020). TRY has previously been successfully used in studies of urban plant communities (Kattge et al., 2011; Zhu et al., 2015). We used Equation 4 plus field measurements of DBH and height of woody stems to calculate the AGB of each woody species identified within each sampling plot. We divided these values by the area of the sampling plot to get the biomass per unit area (kg/m^2^) for each plot.

### 2.9 Multivariate statistics

**FIGURE 5:**
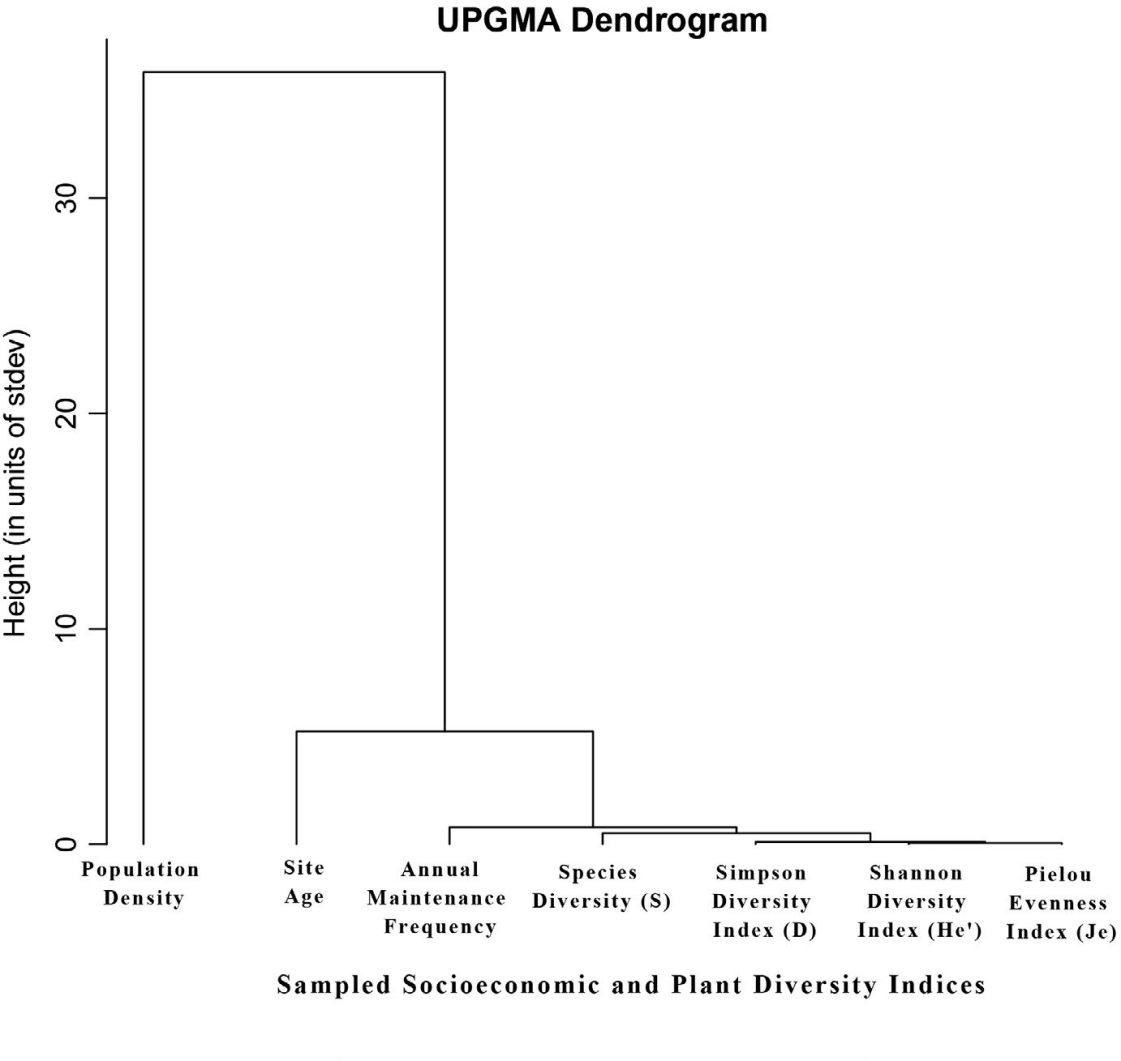
UPGMA tree of dissimilarity using a Euclidean distance matrix of the z‐transformed all variables to detect covariance. Species diversity (*S*), Simpson diversity index (*D*), Shannon diversity index ($H_{e}^{\prime }$), Pielou evenness index (Je), site age, annual maintenance frequency, and population density

We performed multiple regression to determine the combined and individual effects of possible predictors of AGB: species diversity (*S*), Simpson diversity index (*D*), Shannon diversity index ($H_{e}^{\prime }$), Pielou evenness index (Je), site age, annual maintenance frequency, and Population density. We conducted the multiple regression analyses in two ways, with and without variable reduction to account for covariance. We reduced the number of variables using an approach that combines principle component analysis (PCA) with a UPGMA tree (Gao et al., 2020). First, we performed a PCA based on z‐transformations of all variables and generated a UPGMA tree using a Euclidean distance matrix to identify clusters of *z*‐transformed variables. Using the UPGMA tree, we defined highly similar variables as those arising from a node with a height less than 4.0, where heights are roughly in units of standard deviation due to the z‐transformation. For each cluster, we sought to retain the variable with the highest absolute value of loading on PCA axis 1. This resulted in three clusters comprising population density, site age, and all other variables (Figure 5). However, we noted that the large cluster of variables contained annual maintenance frequency, plus the four diversity indices. We decided to retain annual maintenance frequency and perform variable reduction by eliminating all but one diversity index according to the PCA loadings. Thus, our multiple regression analyses with variable reduction comprised the Simpson diversity index (*D*), site age, annual maintenance frequency, and population density in different types of land use as predictors of AGB. We performed both multiple regression analyses and visualized the results in R 4.0.4 (https://cran.r‐project.org/bin/windows/base/).

## 3 RESULTS

The 190 sampled UFUs within Haikou comprised 59 representing the government‐institutional type of land use, 35 representing industrial‐commercial, 12 consisting of park‐recreational, 48 of residential, 33 of transportation infrastructure, and three of undeveloped land (Table 1). Across Haikou, based on the same remote sensing data from SPOT used for sampling design, the total nonwater area is 13651040.62 m^2^, of which 42.90% is vegetation, 53.89% comprises built‐up area, and 3.21% is undeveloped. Our sampling (Table 1) includes only 6.32% samples from parks, allowing those areas of vegetation, or green spaces, to potentially exist within all types of land uses. Among all plots, we detected 173 unique tree species, and the AGB of selected, commonly encountered non‐native species is shown in Table 2. Overall, the 190 UFUs used in this study do not capture the total species diversity of Haikou, which has around 1980 species (Zhu, Pei et al., 2019), and assessing city‐wide diversity is beyond the scope of what we hope to accomplish here.

**TABLE 2:** Aboveground biomass of selected non‐native species

Species Latin Name	Family	Biomass (kg/m^2^)	Biogeographical realm
*Acacia mangium*	Fabaceae	3.41e	Pantropical
*Albizia falcata*	Fabaceae	3.13e	Pantropical
*Archontophoenix alexandrae*	Arecaceae	7.49a	Tropical Asia to Tropical Australasia Oceania
*Corymbia citriodora*	Myrtaceae	5.64b	Tropical Asia to Tropical Australasia Oceania
*Dypsis lutescens*	Arecaceae	7.51a	Tropical Asia to Tropical Africa
*Lagerstroemia speciosa*	Lythraceae	4.45d	Tropical Asia to Tropical Australasia Oceania
*Manilkara zapota*	Sapotaceae	4.43d	Pantropical
*Muntingia calabura*	Muntingiaceae	4.98c	Pantropical
*Pouteria campechiana*	Sapotaceae	4.32d	Pantropical
*Roystonea regia*	Arecaceae	7.21a	Tropical Asia to Tropical Africa
*Schefflera actinophylla*	Araliaceae	4.08d	Pantropical
*Talipariti tiliaceum*	Malvaceae	5.46b	Pantropical
L.S.D (5%)		0.46	

Note

Letters indicate which means are significantly different at the 95% level of confidence.

**FIGURE 6:**
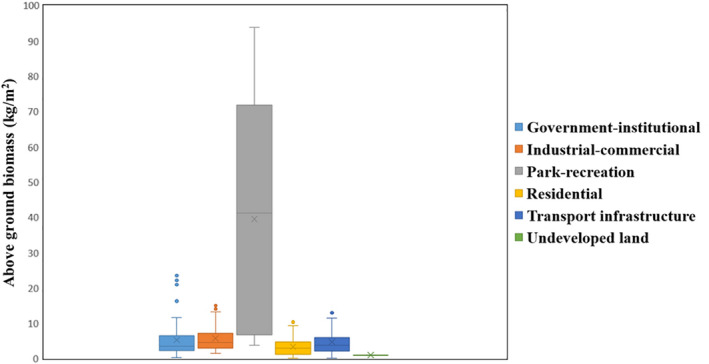
Aboveground biomass per unit area of five urban types of land use

Among types of land use, we found that the highest AGB was within the park‐recreational type (39.49 kg/m^2^), while undeveloped land had the lowest AGB (0.99 k/m^2^). AGB decreased according to the following order of types of land use: park‐recreational, industrial‐commercial area, government‐institutional, transport infrastructure, residential area, and undeveloped land (Figure 6).

The residential and governmental‐institutional land uses had the highest species richness, industrial‐commercial had the highest Simpson diversity index (*D*) and Shannon diversity index ($H_{e}^{\prime }$), and the park‐recreational type of land use had the highest Pielou evenness index (Je). However, after accounting for standard error, the values for all indexes and all types of land uses were largely overlapping. We found that the park‐recreational type of land use had the highest site age (years) and annual maintenance frequency (Table 3).

**TABLE 3:** The mean of Species diversity, Simpson diversity index (*D*), Shannon diversity index ($H_{e}^{\prime }$), Pielou evenness index, site age, annual maintenance frequency, and population density in different types of land use

Land use	Species diversity (*S*)	Simpson diversity index (*D*)	Shannon diversity index ($H_{e}^{\prime }$)	Pielou evenness index (Je)	Site age (years)	Annual maintenance frequency	Population density
Governmental‐institutional	4.24 ± 2.09	0.34 ± 0.06	1.22 ± 0.27	0.82 ± 0.08	48.76 ± 45.28	7.15 ± 3.92	2,576.78 ± 5,127.81
Industrial‐commercial	3.74 ± 1.26	0.36 ± 0.06	1.27 ± 0.17	0.88 ± 0.08	22.34 ± 15.76	5.08 ± 3.28	842.25 ± 880.88
Park‐recreational	3.66 ± 1.45	0.30 ± 0.07	1.02 ± 0.22	0.89 ± 0.10	99.00 ± 116.43	11.18 ± 4.57	1791.25 ± 1,313.89
Residential	4.25 ± 2.63	0.32 ± 0.05	1.19 ± 0.35	0.83 ± 0.08	21.50 ± 28.04	6.31 ± 3.04	1,446.35 ± 802.57
Transport infrastructure	3.82 ± 2.23	0.38 ± 0.08	1.04 ± 0.24	0.78 ± 0.17	30.97 ± 18.08	4.79 ± 3.10	1,081.72 ± 982.84

Based on our multiple regression analysis, we found that the Simpson diversity index (*D*), site age, annual maintenance frequency, and population density were all good predictors of AGB based on the *β* coefficients (−6.54 to 153.31) and *R^2^* values (range: 0.22–0.90) taken together. The Simpson diversity index (*D*) was the strongest predictor of AGB for all types of land uses based on *R^2^* values but showed the greatest strength for the governmental‐institutional type. Notably, *R^2^* values for the multiple regressions were low (i.e., below 6.3) for all types of land use except the park‐recreational type, for which it was very high (0.90). This suggests that most of the variability in AGB can be explained by site age, population density, management frequency, and Simpson's diversity for the park‐recreational type of land use, while other unmeasured factors are affecting AGB in areas representing the other types of land use (Table 4). In industrial‐commercial, park‐recreational, and residential types of land use, the Pielou evenness index (Je) was also an incredibly good predictor of AGB. The Simpson diversity index (*D*) was a good predictor of AGB based on the *β* coefficients (36.44 and 24.22) for governmental‐institutional and transport infrastructure types of land use (Table 5), while site age, annual maintenance frequency, and population density appear to each have a significant positive relationship with the AGB of trees in residential and park‐recreational types of land use (Table 6).

**TABLE 4:** Multiple regression analysis of aboveground biomass (AGB) with Simpson diversity index (*D*), site age, annual maintenance frequency and population density in different types of land use

	*β* coefficient	*SE*	t value	Pr(>|*t*|)	Multiple *R* ^2^	Cohen's *F* ^2^
Governmental‐institutional						
(Intercept)	−5.47	4.62	−1.18	0.24	0.24**	0.31
Simpson diversity index (*D*)	3.00	1.16	2.58	0.01*		
Site age	3.03	1.33	2.26	0.02*		
Annual maintenance frequency	−1.01	1.80	−0.56	0.57		
Population density	−6.54	1.24	−0.52	0.60		
Industrial‐commercial						
(Intercept)	−0.01	4.26	−0.00	0.99	0.22.	0.27
Simpson diversity index (*D*)	21.70	10.34	2.09	0.04*		
Site age	−0.02	0.04	−0.69	0.49		
Annual maintenance frequency	−0.32	0.20	−1.56	0.12		
Population density	0.00	0.00	0.28	0.77		
Park‐recreational						
(Intercept)	−77.34	19.19	−4.02	0.00**	0.90**	9.44
Simpson diversity index (*D*)	153.31	74.88	2.04	0.07.		
Site age	0.19	0.04	4.38	0.00**		
Annual maintenance frequency	2.86	1.00	2.84	0.02*		
Population density	0.01	0.00	3.08	0.01*		
Residential						
(Intercept)	−5.85	2.18	−2.68	0.01*	0.32**	0.48
Simpson diversity index (*D*)	19.93	6.33	3.14	0.00**		
Site age	0.01	0.01	0.97	0.33		
Annual maintenance frequency	0.24	0.12	2.02	0.04*		
Population density	0.000	0.00	1.62	0.11		
Transport infrastructure						
(Intercept)	−3.85	2.62	−1.46	0.15	0.38**	0.62
Simpson diversity index (*D*)	23.17	7.14	3.24	0.00**		
Site age	−0.02	0.02	−0.72	0.47		
Annual maintenance frequency	0.14	0.18	0.81	0.42		
Population density	−0.00	0.00	−0.39	0.69		

Note

Signif. codes: 0 ‘***’ 0.001 ‘**’ 0.01 ‘*’ 0.05 ‘.’ 0.1 ‘ ’ 1.

**TABLE 5:** Multiple regression analysis of aboveground biomass (AGB) with Species diversity, Simpson diversity index (*D*), Shannon diversity index ($H_{e}^{\prime }$), and Pielou evenness index (Je) in different types of land use

	*β* coefficient	*SE*	*t* value	Pr(>|*t*|)	Multiple *R* ^2^	Cohen's *F* ^2^
Governmental‐institutional						
(Intercept)	−31.72	6.57	−4.82	0.00***	0.46***	0.85
Species diversity (*S*)	0.56	0.27	2.04	0.04*		
Simpson diversity index (*D*)	36.44	10.13	3.59	0.00***		
Shannon diversity index ($H_{e}^{\prime }$)	6.952	2.18	3.18	0.00**		
Pielou evenness index (Je)	16.72	7.49	2.23	0.02*		
Industrial‐commercial						
(Intercept)	−28.82	13.55	−2.12	0.04*	0.44**	0.79
Species Diversity (*S*)	1.12	0.52	2.14	0.04*		
Simpson diversity index (*D*)	−8.97	25.60	−0.35	0.72		
Shannon diversity index ($H_{e}^{\prime }$)	9.08	3.70	2.45	0.02*		
Pielou evenness index (Je)	24.92	21.79	1.14	0.26		
Park‐recreational						
(Intercept)	−192.72	24.59	−7.8	0.00***	0.96***	24.00
Species Diversity (*S*)	14.93	2.68	5.57	0.00***		
Simpson diversity index (*D*)	−72.98	74.04	−0.98	0.35		
Shannon diversity index ($H_{e}^{\prime }$)	23.78	20.50	1.16	0.28		
Pielou evenness index (Je)	198.28	34.78	5.70	0.00***		
Residential						
(Intercept)	−13.09	2.76	−4.74	0.00***	0.58***	1.38
Species Diversity (*S*)	0.19	0.11	1.76	0.08.		
Simpson diversity index (*D*)	8.72	6.02	1.44	0.15		
Shannon diversity index ($H_{e}^{\prime }$)	3.55	0.81	4.36	0.00***		
Pielou evenness index (Je)	10.46	3.84	2.72	0.00**		
Transport infrastructure						
(Intercept)	−12.37	2.51	−4.92	0.00***	0.74***	2.85
Species Diversity (*S*)	0.31	0.17	1.76	0.08.		
Simpson diversity index (*D*)	24.22	4.70	5.15	0.00***		
Shannon diversity index ($H_{e}^{\prime }$)	7.84	1.69	4.61	0.00***		
Pielou evenness index (Je)	−1.78	2.18	−0.81	0.42		

Note

Signif. codes: 0 ‘***’ 0.001 ‘**’ 0.01 ‘*’ 0.05 ‘.’ 0.1 ‘ ’ 1.

**TABLE 6:** Multiple regression analysis of aboveground biomass (AGB) with site age, annual maintenance frequency, and population density in different types of land use

	Estimate	*SE*	*t* value	Pr(>|*t*|)	Multiple *R*‐squared	Cohen's *F* ^2^
Governmental‐institutional						
(Intercept)	5.94	1.43	4.15	0.00***	0.15*	0.18
Site age	3.38	1.40	2.41	0.01*		
Annual maintenance frequency	−3.04	1.70	−1.78	0.07.		
Population density	−6.00	1.31	−0.45	0.64		
Industrial‐commercial						
(Intercept)	8.18	1.79	4.55	0.00***	0.10	0.11
Site age	−2.10	4.49	−0.46	0.64		
Annual maintenance frequency	−4.01	2.13	−1.88	0.06.		
Population density	8.88	7.86	0.11	0.91		
Park‐recreational						
(Intercept)	−48.86	15.64	−3.123	0.01*	0.85**	5.67
Site age	0.247	0.045	5.472	0.00***		
Annual maintenance frequency	3.85	1.04	3.68	0.00**		
Population density	0.01	0.00	2.89	0.02*		
Residential						
(Intercept)	0.048	1.22	0.04	0.96	0.17*	0.20
Site age	0.01	0.01	0.79	0.43		
Annual maintenance frequency	0.30	0.13	2.29	0.02*		
Population density	0.00	0.00	1.742	0.08.		
Transport infrastructure						
(Intercept)	3.47	1.53	2.26	0.03*	0.15	0.18
Site age	−0.01	0.03	−0.37	0.71		
Annual maintenance frequency	0.40	0.19	2.13	0.04*		
Population density	−0.00	0.00	−0.46	0.64		

Note

Signif. codes: 0 ‘***’ 0.001 ‘**’ 0.01 ‘*’ 0.05 ‘.’ 0.1 ‘ ’ 1.

## 4 DISCUSSION

### 4.1 Variation in aboveground biomass by types of land use

The AGB within the park‐recreational type of land use of Haikou significantly exceeded that of all other types of land uses in the city, and parks‐recreational areas also had the highest species diversity in terms of Pielou evenness index (Je). In addition to having the highest AGB out of all the urban types of land use, the park‐recreational type of land use experiences the most frequent and extensive management practices. As most park‐recreational land is public (Ramer & Nelson, 2020), this suggests a considerable governmental investment in maintaining these areas that potentially surpasses investments in private land, such as golf courses, by businesses (Furlan & Sinclair, 2021). Thus, this investment seems to yield dividends not only in species richness but also in AGB. However, 20 species were contributing to the majority of the total AGB measured for park‐recreational areas, calling into question whether AGB is dependent on diversity. Nevertheless, out results suggest a relationship between diversity indices and AGB based on the multiple regressions.

Government‐institutional, industrial‐commercial, residential, and transportation infrastructure have lower AGB than parks‐recreational areas. To some degree, the lower AGB in these areas may be due to lower site age and annual maintenance frequency. However, as all R^2^ values AGB and anthropogenic factors for these types of land use were small, aspects such as recent planning that are not reflected by maintenance may be greater contributors to AGB within these areas. For example, planners may prefer small‐statured species for various aesthetic and particle reasons (Threlfall et al., 2016).

### 4.2 Role of non‐native species in AGB within Haikou

Overall, we counted 13 non‐native species among 173 species (7.5%) observed in this study, and these accounted for 0.17%, 0.5%,0.51%, 0.8%, 4%, and of woody individuals observed in transportation infrastructure, industrial‐commercial, government‐institutional, residential, and park‐recreational types of land use. Arguably, non‐native species provide the same ecological functions for the stability of the metropolitan landscape as native species (Cameron & Blanuša, 2016; Chalker‐Scott, 2015; Ramus et al., 2017; Russo et al., 2015; Schlaepfer, 2018), and the exclusion of non‐native species in urban forests is sometimes ill‐advised because native species may not be able to completely fulfill the demands for ecosystem services in large urban settings (Sjöman et al., 2016). For example, citizen groups have lobbied to protect California's non‐native Eucalyptus trees because the trees are important in carbon sequestration (Schlaepfer et al., 2011). In Haikou, the non‐native plant species in the park‐recreational type of land use were likely introduced to meet aesthetic and cultural demands, and they contribute substantially to tree species diversity this type of land use. However, non‐native species are known to contribute less than native species to AGB (Nizamani et al., 2021).

### 4.3 Relationships between aboveground biomass and tree species diversity

Our results indicate that a significant positive linear regression exists between species diversity and AGB across the five urban types of land use within the tropical city of Haikou. These results are consistent with findings from several previous studies that tree species diversity corresponds to high biomass production (Nizamani et al., 2021; Sagar & Singh, 2006; Zhang & Chen, 2015). Maintaining high tree biodiversity in urban areas may limit short‐ and long‐term changes to AGB and help to support a stable urban ecosystem (Li, Lang et al., 2018).

In studies of biodiversity, species represent the common unit of measure, an approach that we took here through the direct measure of species richness as well as several other biodiversity indices fundamentally rooted in counting species. However, the diversity of species indirectly incorporates many other dimensions of biodiversity such as functional, phylogenetic, and genomic diversity, all of which are known to positively impact plant productivity and, consequently, AGB (Naeem et al., 2012). Thus, our results for the relationships of species diversity to AGB may also apply to these other dimensions of tree biodiversity, and this merits future studies.

The Simpson diversity index (*D*) was a good predictor of AGB for all five types of land use (Table 4). In industrial‐commercial, park‐recreational, and residential types of land use, the Pielou evenness index (Je) was also an incredibly good predictor of AGB (Table 5). The Pielou evenness index (Je) considers the similarity of the population sizes of each species within an ecosystem (Pielou, 1966) and, thus, reveals that the biomass of industrial‐commercial, park‐recreational, and residential types of land use increase when they have similar population sizes of each species. The Simpson diversity index (*D*) was a good predictor of AGB for governmental‐institutional and transport infrastructure types of land use (Table 5). The Shannon diversity index ($H_{e}^{\prime }$) reflects the richness and evenness of species (Shannon, 1948); therefore, AGB in these two types of land use increase as both richness and evenness of the community increase These results suggest that areas representing types of land use, such as transportation hubs (e.g., bus stops, airports, train stations), have great potential for increasing urban AGB. While there is rapidly growing global interest in developing such areas as sustainable green spaces (e.g., Hussain & Ramdan, 2020; Kalinke, 2013), the effectiveness of their urban ecosystem services will likely be determined through proper planning that accounts for how different dimensions of diversity drives their AGB.

The relationships between tree species biodiversity and AGB across different types of land use in the tropical and coastal city of Haikou may have explanations rooted in classic ecological theory. For example, species co‐existence, and thus higher tree species diversity, within urban green spaces may be facilitated by resource input through greater management, especially within the park‐recreational type of land use. On the other hand, urban green spaces may be governed by different principles in some cases, such as the case of undeveloped land, which, despite representing an open, highly disturbed area, has not recruited much or any woody vegetation in Haikou in contrast to recruitment in areas, such as light gaps or burned patches in nature (Walters et al., 2020).

Nevertheless, tree species diversity in urban spaces is also strongly linked to human preference, and some of these preferences are motivated by costs. For example, planting a lower diversity of species usually carries lower short‐term maintenance costs (Huang et al., 2021). However, lower diversity also may lead to the lower stability of the urban ecosystem in the long term. In particular, low diversity green spaces could be devastated by disease within a few years (Horváthová et al., 2021), such as when Dutch elm disease (*Ophiostoma ulmi*) eliminated millions of American elm trees (*Ulmus americana*), which were widely planted along North American roads. Increased diversity among tree communities reduces the risk of pest, disease, and insect pest damage, as well as environmental changes (Jernelöv, 2017), but necessitates those planners to take a long view on costs of urban green space development.

### 4.4 Anthropogenic drivers (site age, annual maintenance frequency, and population density) of AGB in urban areas

While site age, annual maintenance frequency, and population density appear to each have a significant positive relationship with the AGB of trees, site age explains more of the variation in AGB in residential and park‐recreational types of land use (Table 4, Table 6). This is consistent with findings in other temperate, coastal cities in China (Wang et al., 2020). An older site age may allow more time for vegetation growth or better facilitate recruitment of native or naturalized species capable of generating high biomass within the local environment (Cheng et al., 2018; Dostál et al., 2013; Li et al., 2018; Ossola & Hopton, 2018).

Although site age appears to be the strongest driver of AGB in Haikou among anthropogenic drivers, we also discovered a significant positive relationship between AGB and annual maintenance frequency for the park‐recreational types of land use. Prior studies in other areas of the world have also shown that management practices are important for maintaining the high AGB of tree species, such as in cities throughout Zambia (Pelletier et al., 2017).

Human population density has a significant positive relationship with the AGB of trees and, by extension from our data, likely also impacts tree species diversity. Notably, human preferences for certain species due to both cultural (e.g., banyan) and tangible (e.g., mango) benefits have likely imposed selection effects on urban tree assemblages (Blicharska & Mikusiński, 2014), and the effects of these preferences may be higher in areas with higher population density like a governmental‐institutional and residential areas. Within a local plant species pool, species with tangible and cultural values have often been selectively maintained over less desirable species (Huang et al., 2020). Species that are deliberately selected and put under conscious human care are an integral component of the tree biodiversity of Haikou and AGB. However, high population density may also negatively impact the prevalence of high‐yielding spontaneous vegetation, which humans may regard as weeds and remove.

In general, sites bearing spontaneous vegetation could be substantially older than any buildings that surround them. Over time, the relative frequency of cultivated green space within a city will typically increase compared to spontaneous green spaces, which will decrease (Riley et al., 2018). The distinctions between these types of green space are very important and likely have profound impacts on tree species diversity and thus AGB. While we did not differentiate between spontaneous and cultivated green space in this study, it merits future work.

## 5 CONCLUSIONS

We discovered a significant positive relationship between AGB and tree species diversity, especially based on Simpson's D across four types of land use (government‐institutional, industrial‐commercial, park‐recreational, residential, and transportation infrastructure) in the tropical city of Haikou, China. The relationship was the strongest for park‐recreational areas, which constitute the largest portion of green space within the city. Furthermore, we found that the relationships between tree AGB and anthropogenic drivers of green space, site age, population density, and annual maintenance frequency were significantly positively correlated, highlighting the role of human input in fostering urban green space in Haikou. Site age was the strongest anthropogenic driver of AGB, but maintenance frequency was also very important, especially in park‐recreational areas. This suggests that both legacy and maintenance effects, which are known to drive the distributions of green space overall in urban areas, also significantly impact AGB. Future comparative studies among cities may utilize our standardized field data collection protocols, while continued studies within Haikou and elsewhere may shed light on the differing roles and distributions of spontaneous and cultivated urban green space.

## CONFLICTS OF INTEREST

The authors declare no conflicts of interest.

## AUTHOR CONTRIBUTION

**Mir Muhammad Nizamani:** Conceptualization (equal); Data curation (lead); Formal analysis (lead); Investigation (lead); Methodology (equal); Software (lead); Validation (equal); Visualization (lead); Writing‐original draft (lead); Writing‐review & editing (equal). **AJ Harris:** Conceptualization (equal); Data curation (supporting); Formal analysis (equal); Investigation (equal); Methodology (equal); Software (equal); Validation (equal); Visualization (equal); Writing‐original draft (equal); Writing‐review & editing (lead). **Xia‐Lan Cheng:** Formal analysis (equal); Investigation (equal); Methodology (equal); Software (equal); Writing‐original draft (supporting); Writing‐review & editing (equal). **Zhi‐Xin Zhu:** Funding acquisition (equal); Methodology (equal); Project administration (equal); Visualization (equal); Writing‐review & editing (equal). **Chi Yung Jim:** Writing‐review & editing (equal). **Hua‐Feng Wang:** Conceptualization (lead); Data curation (supporting); Formal analysis (equal); Funding acquisition (lead); Investigation (lead); Methodology (lead); Project administration (lead); Resources (lead); Software (equal); Supervision (lead); Validation (lead); Visualization (equal); Writing‐original draft (supporting); Writing‐review & editing (lead).

## Data Availability

All data used in this paper are included as Appendix 1.
